# Susceptibility to Predation Affects Trait-Mediated Indirect Interactions by Reversing Interspecific Competition

**DOI:** 10.1371/journal.pone.0023068

**Published:** 2011-08-17

**Authors:** Sophie L. Mowles, Simon D. Rundle, Peter A. Cotton

**Affiliations:** 1 Marine Biology and Ecology Research Centre, The University of Plymouth, Plymouth, United Kingdom; 2 School of Biosciences, The University of Nottingham, Sutton Bonington Campus, Loughborough, United Kingdom; University of British Columbia, Canada

## Abstract

Numerous studies indicate that the behavioral responses of prey to the presence of predators can have an important role in structuring assemblages through trait-mediated indirect interactions. Few studies, however, have addressed how relative susceptibility to predation influences such interactions. Here we examine the effect of chemical cues from the common shore crab *Carcinus maenas* on the foraging behavior of two common intertidal gastropod molluscs. Of the two model consumers studied, *Littorina littorea* is morphologically more vulnerable to crab predation than *Gibbula umbilicalis*, and it exhibited greater competitive ability in the absence of predation threat. However, *Littorina* demonstrated a greater anti-predator response when experimentally exposed to predation cues, resulting in a lower level of foraging. This reversed the competitive interaction, allowing *Gibbula* substantially increased access to shared resources. Our results demonstrate that the susceptibility of consumers to predation can influence species interactions, and suggest that inter-specific differences in trait-mediated indirect interactions are another mechanism through which non-consumptive predator effects may influence trophic interactions.

## Introduction

Predator-prey interactions are fundamental aspects of ecology, yet it is comparatively recently that non-lethal interactions and the indirect effects of predators have been considered in detail. Assemblages of interacting species have long been conceptualized as food webs, where species interact with one another via consumer–resource links. Chains of such direct interactions can also allow species to interact indirectly with one another through one or more intervening species [1], [2]. The trophic cascade concept incorporates such indirect effects brought about by density changes in species groups, termed ‘density-mediated indirect interactions’ (DMIIs, [3]). The result is an oscillating relationship in the strength of interactions between different trophic levels. In a simple three-level system, for example, when predation increases, prey density will be reduced, thus alleviating grazing pressure on the prey's resource [3]. A predator can therefore have an indirect effect on the abundance of primary producers by regulating herbivore density via consumption [4], [5].

The trophic cascade concept has been widely accepted, and indirect interactions between organisms were originally thought to have been initiated through these density-mediated routes alone. For example, classic studies by Paine [6], [7] illustrated the disproportionate effect that the predatory starfish *Pisaster ochraceus* had on populations of the mussel *Mytilus californianus*. Paine [8] argued that such keystone predators must influence the density of their prey through preferential consumption, but recent research suggests that non-consumptive effects may underlie predator mediated prey coexistence [9]. Indeed, growing evidence demonstrates that predators can also interact with prey by inducing modifications in prey phenotype, which can alter interactions with other species in the system [1]. Such phenotypic responses, termed ‘trait-mediated interactions’ (TMIs, [3]) or more simply, ‘non-consumptive effects’, can be at the level of development, morphology, physiology, life history and behavior [10], [11] and may cascade down to affect the prey's resources (trait-mediated indirect interactions, or TMIIs). Recent analyses indicate that trait-mediated effects are generally as strong, or stronger, than the effects of direct consumption [12]–[15]. Non-consumptive effects appear to be particularly prevalent in aquatic ecosystems and have marked effects on foraging efficiency and foraging effort [14].

Where prey undertake anti-predatory responses, they invariably incur some cost. For example, vulnerability to predators usually increases whilst prey forage, but avoiding predators often means decreasing food intake [16]. Alternatively, prey organisms may be forced to migrate to poor-quality habitats with a lower predation risk (e.g., [17]). Both of these responses result in the same outcome: the prey suffer a reduction in overall resource acquisition, which results in a trophic cascade affecting the abundance and/or diversity of the resource (e.g., [5]). At the same time, there may be implications of such non-consumptive effects on competitive interactions within food chains. For example, Relyea [18] demonstrated that the presence of a predator could reverse competition in amphibian larvae through species differences in morphological plasticity. Similarly, predator effects have been shown to be modified by prey size, both within [19] and between species [1], [20].

Whereas morphological plasticity and ontogenetic changes in body size differences may reduce the probability of predation during an encounter, behavioral avoidance serves to reduce encounter rates. Furthermore, behavioral avoidance can occur immediately on encountering a predator whilst undergoing morphological changes requires a considerably longer time period. Habitat shifts and reductions in feeding rate in response to a predator have been documented in a wide range of studies (reviewed in [15]), and in many cases predator avoidance leads to an overall reduction in grazing, which in turn leads to a TMII in which producer biomass increases. Indeed, the strength and prevalence of foraging–predation risk trade-offs led Schmitz et al. [15] to suggest that these are the ultimate mechanism behind trophic cascades.

**Figure 1:**
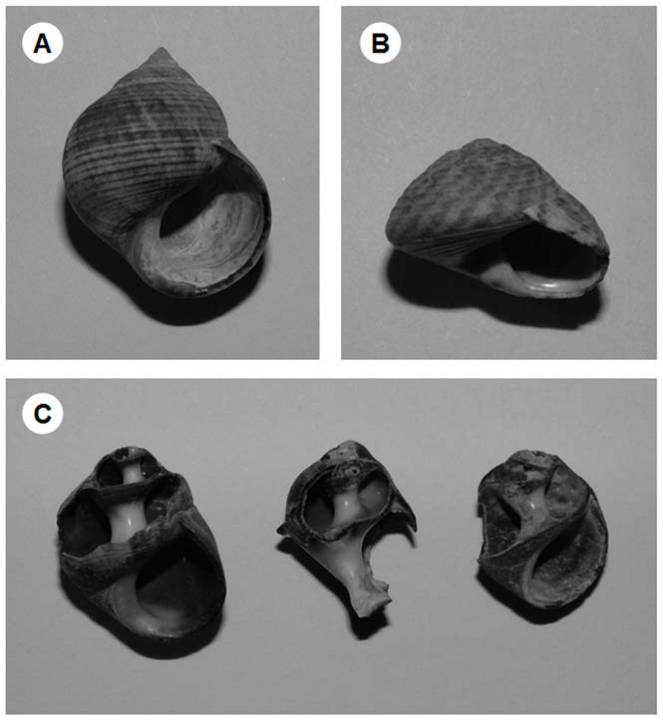
A comparison of the morphologies of the model prey species used in the study. A) The common periwinkle *Littorina littorea* displays a high aspect ratio; B) the flat topshell *Gibbula umbilicalis* displays a low aspect ratio (discoid morphology); and C) evidence of crab predation on *L. littorea*.

Aquatic gastropods exhibit a wide range of adaptive morphologies as protection from predators. Clearly shell strength is an important defense [21], [22] and several gastropod species have been shown to exhibit inducible increases in shell thickness in response to waterborne risk cues from predators such as crabs and fish [5], [23]–[29]. However, shell structure can also provide an effective form of defence [22]. For example, shells that are highly polished or have a more discoid shape can be more difficult for predators such as crayfish and crabs to handle [30]. However, gastropods can also show a trade-off, or trait compensation, between morphological and behavioral defences [31], [32]. Cotton et al. [30] demonstrated a negative correlation between morphological defences and behavioral avoidance strategies across four species of intertidal gastropod; species with high aspect ratios (tall spires) were most vulnerable to predation (common periwinkle *Littorina littorea*, Fig. 1A) and compensated by showing the highest level of behavioral avoidance, while the species with low aspect ratios (short spires: discoid shells) were least vulnerable to predation (flat topshell *Gibbula umbilicalis*, Fig. 1B) and showed the lowest responsiveness.

Here we chose to use these two extremes of trait expression as a model system for examining how susceptibility to predation can influence non-consumptive effects. In the first experiment we tested the *a priori* prediction that *Gibbula* (less susceptible) would respond to predation cues less than *Littorina* (more susceptible to predation by crabs, Fig. 1C) and that *Gibbula* would therefore continue grazing to a greater extent than *Littorina* when exposed to predation cues. In the second experiment we assessed the competitive ability of these two gastropod species in the presence and absence of predation cues. As competitive interactions between intertidal gastropods may be related to their susceptibility to predation, our second prediction was that the morphologically vulnerable species (*Littorina*) would be at a competitive disadvantage and, hence, graze relatively less algal resource, under predation cue conditions.

## Methods

### Study organisms

All animal work was carried out in accordance with the ASAB/ABS Ethical Guidelines. Approval was not necessary for this work as it involves gastropod molluscs and decapod crustaceans; invertebrates which require no research permits or approval in the UK. Likewise, we did not require permits to access the collection sites, nor to remove animals and algae for laboratory studies as the collection sites were on public land that was not National Park land or Government Protected; and there is no legislation restricting the collection of these organisms. Gastropods used in the production of predation cues were first humanely killed by freezing. To minimize the number of individuals used we kept sample sizes to the minimum suitable for effective statistical analysis. Furthermore, those individuals used in the trials were kept for a short time in optimal conditions before being returned to the shore.

We collected *Littorina littorea* (L.) and *Gibbula umbilicalis* (da Costa) from Hannafore Point, UK (50° 20′N, 4° 27′W). The snails were standardized to a small size range for all experiments (average width 11.95 mm; range 10.7–12.5 mm) and were maintained on sea lettuce (*Ulva lactuca* L.) collected from Plymouth Hoe, UK (50° 22′N, 4° 08′W). There was no significant species difference in shell width (Experiment 1: *t* = 0.821, d.f. = 118, p>0.05; Experiment 2: *t* = 0.516, d.f. = 118, p>0.05), but *Gibbula* had a significantly lower aspect ratio than *Littorina* (Experiment 1: *t* = 49.51, d.f. = 118, p<0.0001; Experiment 2: *t* = 29.99, d.f. = 118, p<0.001).

Shore crabs (*Carcinus maenas* L.) were collected from the Plym Estuary, UK (50° 22′N, 4° 07′W) and Heybrook Bay, UK (50° 19′N, 4° 07′W) and were maintained on a diet of common mussels (*Mytilus edulis* L.). Overall, nine male crabs were used to prepare the predation cue water. All of the crabs were undamaged and relatively large (average carapace width 60.5 mm; range 55.5–68.2 mm). Throughout the experiment all study organisms were maintained in aerated seawater (35 PSU) at 15°C.

### Experiment 1 – Single species trials

This experiment followed a similar protocol to Cotton et al. [30]. Sixty snails of each species were food deprived for 48 hours and then assigned to one of two treatments (control or predation cue). The 48 hour period of food deprivation ensured that each focal individual was equally motivated to graze on the algal resource provided during the experimental trials. An individual snail was then placed into a circular plastic dish (diameter 157 mm), filled to a depth of 35 mm with seawater (680 ml) and allowed to acclimate for one hour prior to the trial. At the start of each trial a disc of *Ulva* (diameter 23 mm, area 415 mm^2^) was positioned at the centre of the dish and 68 ml of control (normal seawater) or predation cue was added. Predation cue water was made separately for each gastropod species and was taken from two aquaria each containing 1700 ml of seawater in which one *Carcinus* had been maintained for 24 hours. Two snails conspecific to the test species were fed to each crab at the onset of the 24 hour period and an additional crushed conspecific snail was added to the tank immediately before the trials. This preparation exposed the snails to both chemical cues from natural predators, alarm cues from conspecifics, and effluent from crushed conspecifics. The mixture provided the prey with several olfactory cues (predator kairomones and prey death pheromones), a combination that was chosen in order to maximize anti-predator behavior as it indicates that predation is actually occurring [28], [30], [33]. While the individual dishes contained stationary water, any saturation of predation cue would be analogous to that experienced by prey sharing a rock pool with a predator during low tide.

Behavioral data were collected by scan sampling at 15 minute intervals for the first 300 minutes. Behavior was scored as: Eating, Moving, Stationary, At waterline/Out of water. Fleeing to the waterline and exiting the water are well-documented anti-predatory behaviors performed by gastropods [34]–[36]. The overall amount of foraging was quantified from the area of *Ulva* grazed. After 24 hours, the *Ulva* disks were removed, placed between a sheet of clear acetate and graph paper, and then scanned into PhotoStudio 5. The area (mm^2^) of the remaining algae was then calculated.

### Experiment 2 – Two species trials

This experiment followed a protocol similar to Experiment 1. Sixty snails of each species were food deprived for 48 hours and were then assigned to one of two treatments (control or predation cue) in conspecific (*Littorina-Littorina*, *Gibbula-Gibbula*) or heterospecific (*Littorina-Gibbula*) pairs. Pairs of gastropods were then placed in each dish before the addition of *Ulva* and the control or predation cue. Three different predation cues were prepared by feeding a single *Carcinus* with two snails (corresponding to the treatment pairing, e.g., *Littorina-Gibbula*), and adding two crushed snails of the same species immediately prior to the experiment.

Behavioral and foraging data were collected as for Experiment 1, but because of limitations on the availability of unblemished sheets of *Ulva* in October, the disks used were smaller (diameter 13 mm, area 133 mm^2^). In order to ensure that these disks were not entirely consumed, trials in Experiment 2 ran for 195 mins. Behavioral data in these trials can be quantified for each individual (although they are statistically non-independent), but the amount of grazing could only be quantified per trial, not per individual snail.

### Statistical analysis

Statistical analyses were performed using SPSS 15. Throughout we have used the difference between predation cue and control groups as a measure of effect size (see [30], [32]). This is important as a means of standardization because gastropod species differ in their background levels of movement and foraging behavior.

We used non-parametric analysis where possible because of significant heterogeneity of variance that could not be rectified through transformations. The results of Experiment 1 were analyzed using Mann-Whitney tests, and the amount of grazing in Experiment 2 using Kruskal-Wallis tests. Behavioral data in Experiment 2 were non-independent within trials, requiring the use of a repeated measures design. We used the Repeated Measures General Linear Model in SPSS as there is no non-parametric equivalent to a two-factor repeated measures ANOVA. Data for time out of water passed Levene's test for heterogeneity of variance, but the data for time spent eating could not be transformed to achieve homogeneity. However, Underwood [37] considers large, balanced ANOVA designs to be robust to heterogeneity of variance and unlikely to lead to a Type 1 error.

## Results

### Experiment 1 – Single species trials

**Figure 2:**
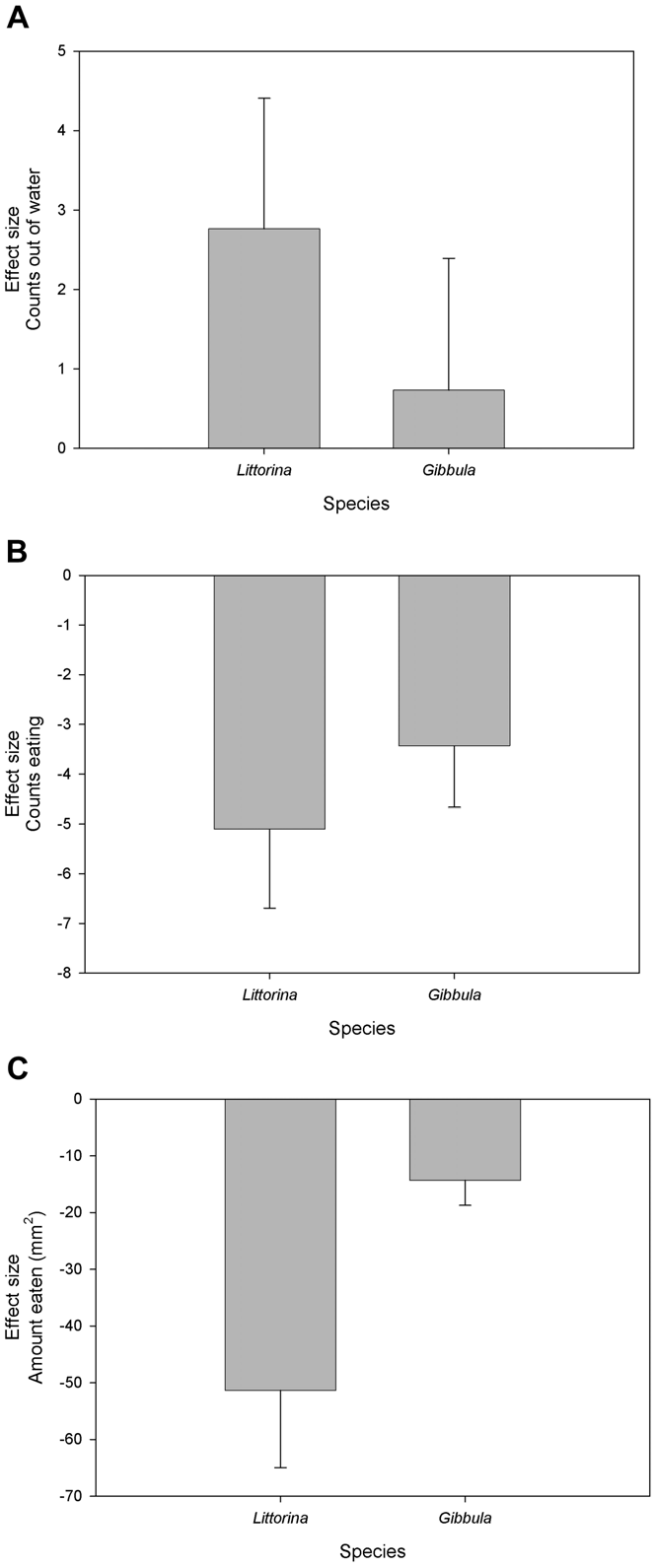
Interspecific comparisons (mean ± 1 SE) between *Littorina littorea* and *Gibbula umbilicalis* from single species trials. The values are means, standardized with respect to the control, for each species. Negative values indicate a reduction relative to the control while positive values indicate an increase relative to the control. A) the number of scans in which an individual was recorded out of the water in predator avoidance (U = 300, d.f. = 58, p<0.025); B) the number of scans in which an individual was recorded eating *Ulva* (U = 301, d.f. = 58, p<0.025); and C) the amount of *Ulva* eaten at the end of the trial (U = 234, d.f. = 58, p<0.001).

We found a significant species difference in the main anti-predator response, climbing to the waterline or out of the water. The addition of predation cue caused *Littorina littorea* to spend significantly more time out of the water than did *Gibbula umbilicalis*, which showed very little standardized response to the predator treatment (U = 300.0, d.f. = 58, p<0.025; Fig. 2A). As a result of spending more time in predator avoidance during predation cue trials, *Littorina* spent significantly less time feeding than *Gibbula* (U = 301.0, d.f. = 58, p<0.025; Fig. 2B). Consequently, the predator treatment reduced the amount of *Ulva* eaten by *Littorina* in 24 hours by over 54%, resulting in *Littorina* showing a significantly greater effect size than *Gibbula* (U = 234.0, d.f. = 58, p<0.001; Fig. 2C).

### Experiment 2 – Two species trials

**Figure 3:**
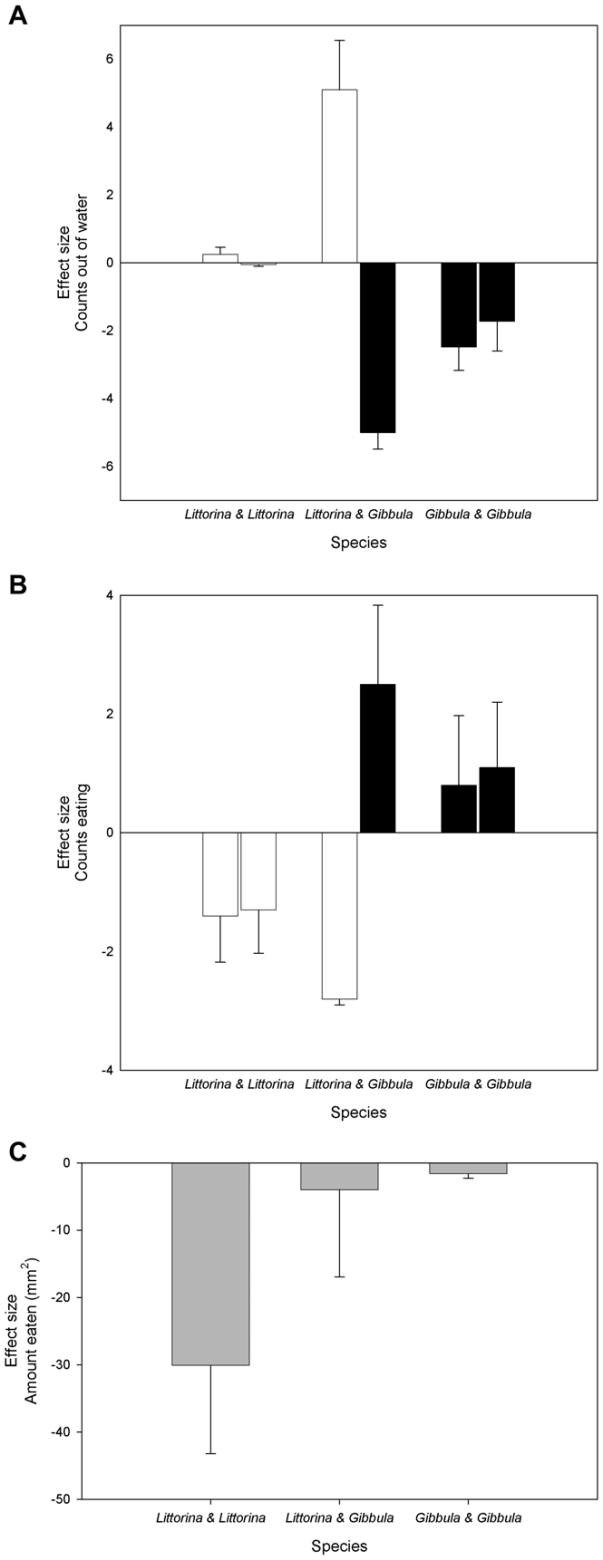
Comparisons (mean ± 1 SE) between *Littorina littorea* (white bars) and *Gibbula umbilicalis* (black bars) from trials with conspecific and heterospecific pairs. The values are means, standardized with respect to the control, for each species. Negative values indicate a reduction relative to the control while positive values indicate an increase relative to the control. A) the number of scans in which each individual was recorded out of the water in predator avoidance (GLM repeated measures: within factors Trial*Treatment, F_2,27_ = 4.141, p<0.03; between factors Treatment F_2,27_ = 3.35, p<0.05); B) the number of scans in which each individual was recorded eating *Ulva* (GLM repeated measures: within factors Trial*Treatment, F_2,27_ = 24.918, p<0.0001; between factors Treatment F_2,27_ = 6.055, p<0.007); and C) the amount of *Ulva* eaten at the end of the trial (K-Ω *χ* ^2^_2_ = 8.476, p<0.015). Bars in C are shown in grey as it was not possible to determine the amount of *Ulva* eaten by each individual.

As in Experiment 1, *Littorina* showed a far more marked response than *Gibbula* to the predation cue. Within trials involving *Littorina* and *Gibbula* pairs, under control conditions *Littorina* typically displaced *Gibbula* from the food and *Gibbula* generally responded to displacement by leaving the water. This situation reversed with the addition of predation cue (Fig. 3A), and the standardized time spent out of the water shows a significant interaction of treatment with the experimental species pairings (GLM repeated measures: within factors Trial*Treatment, F_2,27_ = 4.141, p<0.03; between factors Treatment F_2,27_ = 3.35, p<0.05). In conspecific pairs, *Littorina* showed little effect of the addition of predation cue (Fig. 3A), which is puzzling given the marked response in Experiment 1. It appears that this lack of effect is caused by one individual *Littorina* displacing the other from the algal resource during control trials, leading to an elevated level of crawl-out behavior in the absence of predation cue. *Gibbula* in conspecific pairs showed a slightly reduced level of crawl out behavior in predation cue trials, indicating as above that they do not show high levels of avoidance to the predator treatment.

The results of the foraging activity (Fig. 3B) almost mirror those of the crawl-out behavior. In conspecific pairs, *Littorina* showed depressed foraging activity in the predator treatment while *Gibbula* pairs showed slightly elevated foraging relative to control conditions. The most marked effect was the highly significant trial*treatment interaction, in which *Littorina-Gibbula* trials showed a marked species difference; *Littorina* showed a reduction in foraging behavior compared to controls whereas *Gibbula* increased foraging time relative to the control (GLM repeated measures: within factors Trial*Treatment, F_2,27_ = 24.918, p<0.0001; between factors Treatment F_2,27_ = 6.055, p<0.007).

In the two species trials it was not possible to distinguish the relative grazing by the two individuals. However, we found a significant difference between species-pairs in the relative amount eaten in each treatment (K-W *χ* ^2^_2_ = 8.476, p<0.015; Fig. 3C). Within trials involving *Littorina* pairs, the amount of grazing in the presence of predation cue was greatly reduced compared with controls, while *Gibbula* pairs and heterospecific pairs showed little effect of the predator treatment.

## Discussion

Recent studies have shown that non-consumptive effects may significantly affect trophic cascades within rocky intertidal assemblages. The presence of predatory crabs and fish suppresses grazing by consumers such as gastropods, amphipods and isopods, resulting in a greater density and biomass of algae [5], [38], [39]. Similarly, predatory crabs have been shown to suppress predation of barnacles by the gastropod *Nucella lapillus* [5], [40], [41]. Our study demonstrates that susceptibility to predation may modify interspecific competitive interactions within intertidal food chains, potentially increasing or decreasing the effect of a predator in inducing TMIIs. In our model system of two marine gastropods differing in morphological susceptibility to predation, susceptibility was linked to their behavioral response to predation risk which, in turn, influenced their grazing rate and their interaction with one another. The results supported our *a priori* hypothesis, that *Littorina littorea*, with its higher aspect ratio and greater susceptibility to predation [30] would display a greater degree of responsiveness to predation cues, thus showing a larger reduction in feeding under predation cue conditions than the more discoid *Gibbula umbilicalis*.

In response to predation cues, *Littorina* exhibited both suppressed feeding and anti-predator avoidance behavior, whereas *Gibbula* showed little response. These findings are consistent with those of Cotton et al. [30], which clearly demonstrated a difference between these two species in their handling times by *Carcinus maenas*. The major morphological difference between the species is their contrasting aspect ratios, which affects their resistance to predation by crabs as conical shells with larger aspect ratios are easier to handle than discoid shells with smaller aspect ratios [30]. Furthermore, the results of our second experiment demonstrate that the behavioral responses of individual species influence interspecific interactions, with a reversal in competition and foraging success in the presence of predation cues. *Gibbula* was displaced from the food when in competition with *Littorina*, but when cues from predatory crabs were present *Littorina* showed an anti-predator habitat shift that allowed *Gibbula* greater access to resources.

The presence of predators has been demonstrated to influence, and indeed reverse, competitive interactions between prey species through non-consumptive effects. Relyea [18] showed that the outcome of competitive interactions between wood frog (*Rana sylvatica*) and leopard frog (*R. pipiens*) tadpoles was reversed in the presence of predatory dragonfly larvae (*Anax* spp.); largely through the effects of predator-induced morphological plasticity. Similarly, Peacor and Werner [42] demonstrated that non-consumptive effects affect competition between small and large bullfrog (*R. catesbeiana*) tadpoles. Our experiments demonstrate similar predator effects on competition, but mediated through species differences in their behavioral responses to predation cues, which are potentially influenced by differences in predation susceptibility [30].

With the increasing recognition of the importance of the non-consumptive effects of predators it is becoming clear that many effects originally attributed to direct effects may in fact be the result of non-consumptive indirect effects [9]. For example, in the context of our study the classic work of Lubchenco [43] was considered a textbook example of a density-mediated trophic cascade (e.g., [44], [45]). Lubchenco demonstrated that predation by *Carcinus* indirectly influences rocky shore algal community structure and diversity by controlling *Littorina* density, however, the non-lethal effects of *Carcinus* on herbivorous snails can generate similar outcomes (empirically tested by Trussell et al. [46] and reviewed in Peckarsky et al. [9]). In order to recognise how such non-lethal effects may manifest, Preisser and Bolnick [14] recently used the Lotka-Volterra predator-prey model as a heuristic tool for understanding the impact of non-consumptive effects on prey populations. Within this context, predators may affect prey numbers indirectly by altering their foraging efficiency or their foraging effort. In general, prey should forage in habitats with the lowest ratio of mortality (μ) to foraging rate (f) the “μ/f rule” [47]; when profitable habitats have a high risk of predation, prey may choose less energetically rewarding habitats with lower risk. Alternatively, the presence of a predator may cause prey to leave the habitat temporarily, for example the crawl-out response of gastropods [32], [33]. Both non-consumptive effects reduce net food intake, but our results indicate that the extent to which this occurs depends on susceptibility to predation.

In our experiments *Littorina* was unable to relocate to forage in a low risk habitat, but frequently left the water to avoid predation, reducing overall food intake. *Gibbula*, in contrast, are less susceptible to crab predation and so following Gilliam and Fraser [47] can be considered to be less sensitive to μ. In the presence of predation cues *Gibbula* continued to forage, and indeed in interspecific competition they benefitted from predation cues as *Littorina* departed. Our experiments clearly demonstrate an interaction modification in which an inferior competitor temporarily gains the upper hand as a result of a non-consumptive predator effect. This result highlights one of the many complex ways in which predators may influence the populations of their prey and of their primary resources, and strengthens the argument for further incorporating non-consumptive effects into the study of food-web dynamics as suggested by Peckarsky et al. [9].
